# Causal relationship between osteoarthritis with atrial fibrillation and coronary atherosclerosis: a bidirectional Mendelian randomization study of European ancestry

**DOI:** 10.3389/fcvm.2023.1213672

**Published:** 2023-07-31

**Authors:** Meng Yin, Wenchang Xu, Jixiang Pang, Siwen Xie, Mengting Xiang, Bin Shi, Hua Fan, Gongchang Yu

**Affiliations:** ^1^ Shandong Academy of Occupational Health and Occupational Medicine, Shandong First Medical University & Shandong Academy of Medical Sciences, Jinan, China; ^2^ School of Acupuncture and Tuina, Shandong University of Traditional Chinese Medicine, Jinan, China; ^3^ Neck-Shoulder and Lumbocrural Pain Hospital of Shandong First Medical University, Shandong First Medical University & Shandong Academy of Medical Sciences, Jinan, China; ^4^ Department of Development Planning and Discipline Construction, Shandong First Medical University & Shandong Academy of Medical Sciences, Jinan, China

**Keywords:** osteoarthritis, atrial fibrillation, coronary atherosclerosis, cardiovascular diseases, bidirectional Mendelian randomization, genome-wide association study, European

## Abstract

**Background:**

Osteoarthritis (OA) is a degenerative disease with high prevalence. Some observational studies have shown that patients with osteoarthritis often have co-existing cardiovascular diseases (CVD) such as atrial fibrillation (AF) and coronary atherosclerosis (CA). However, there is still a lack of stronger evidence confirming the association between osteoarthritis and cardiovascular disease. In this study, we used a bidirectional two-sample Mendelian randomization study to investigate the relationship between OA with AF and CA.

**Methods:**

OA data from the UK Biobank and arcOGEN (Arthritis Research UK Osteoarthritis Genetics, a study that aimed to find genetic determinants of osteoarthritis and elucidate the genetic architecture of the disease) integration were selected for the study (*n* = 417,596), AF data were obtained from six studies (*n* = 1,030,836), and coronary atherosclerosis data were derived from the FinnGen (*n* = 218,792). MR analysis was performed primarily using the Inverse variance weighted (IVW) method, with MR Egger, weighted median, simple mode, weighted mode as supplements, sensitivity analysis was performed using Cochran Q statistic, and leave-one-out analysis.

**Results:**

We found that OA and AF were positively associated [IVW: OR (95% CI): 1.11 (1.04, 1.19), *P* = 0.002], while OA and CA were negatively associated [IVW: OR (95% CI): 0.88 (0.79, 0.98), *P* = 0.02]. In the reverse MR analysis, no effect of AF on OA was found [IVW: OR (95% CI): 1.00 (0.97, 1.03), *P *= 0.84], meanwhile, CA and OA were found to be associated negatively [IVW: OR (95% CI): 0.95 (0.92, 0.99), *P *= 0.01]. No violations of MR assumptions were found in the sensitivity analysis.

**Conclusion:**

This research confirms that OA is a risk factor for AF, and there is a mutual protective factor between OA and CA. However, further studies are still necessary to elucidate the underlying mechanisms.

## 1. Introduction

Osteoarthritis is a common degenerative joint disease mainly caused by cartilage injury (1). Established risk factors for osteoarthritis include age, obesity, inflammation, trauma, and genetic factors (2). The prevalence of OA is increasing with the global aging population. It is estimated to affect 250 million people worldwide (3).

Cardiovascular disease (CVD), one of the primary causes of death in the global population, includes both heart and vascular diseases such as arrhythmias and coronary artery disease (CAD) (4). As one of the most frequent arrhythmias, atrial fibrillation (AF) is a major risk factor for death, stroke and other diseases, and the incidence is on the rise over the years (5). According to the Framingham Heart Study (FHS), the incidence of AF has increased threefold over the past 50 years (6). Palpitations, weakness, dizziness and breathlessness are the main clinical symptoms of atrial fibrillation (7). Age is a significant risk factor for atrial fibrillation, with a prevalence of up to 10% in people over the age of 75, which is the same risk factor as for osteoarthritis. Atherosclerotic coronary artery disease is the leading cause of death from cardiovascular disease, accounting for almost 45% of all cases (8). The main clinical symptom of coronary atherosclerosis is exertional angina, sometimes with chest tightness. It's also common in the elderly (9).

In an observational study, arthritis is found to be a common comorbidity in patients with CVD [20.9% (19.5%–23.5%)] (10). A meta-analysis shows that OA is related to an increased risk of cardiovascular disease (11). Although epidemiology has linked OA and CVD, confounders such as age, obesity, sex, use of NSAIDs, and inflammation may affect the conclusions of observational studies. Furthermore, for both ethical and practical reasons, the relationship between OA and CVD cannot be verified by randomized clinical trials (12). Thus, the true causal relationship is still unclear.

Genome-Wide Association Study (GWAS) is a multicenter, large-sample, iterative validation study of gene-disease associations at the genome-wide level (13). Mendelian Randomization (MR) is an experimental method widely used in epidemiology to analyze the causal relationship between exposure and outcome factors by introducing an instrumental variable (iv) as an intermediate variable (14). Based on Mendel's law of independent distribution, gene-disease associations are not confounded by common confounders such as postnatal environment, socioeconomic status, behavioral factors, or reverse causality, and estimates of effects are much closer to the true picture (15). With the lack of randomized clinical trials, Mendelian randomization is an essential method for making inferences about causality.

Based on existing genetic databases, genetic variants controlling the pathogenesis of OA were used as instrumental variables to further investigate the effect of OA on the risk of developing atrial fibrillation and coronary artery disease. Our study aimed to elucidate the potential contribution of OA to both diseases at the genetic level by a two-sample MR method.

## 2. Materials and methods

### 2.1. Methods

**Figure 1:**
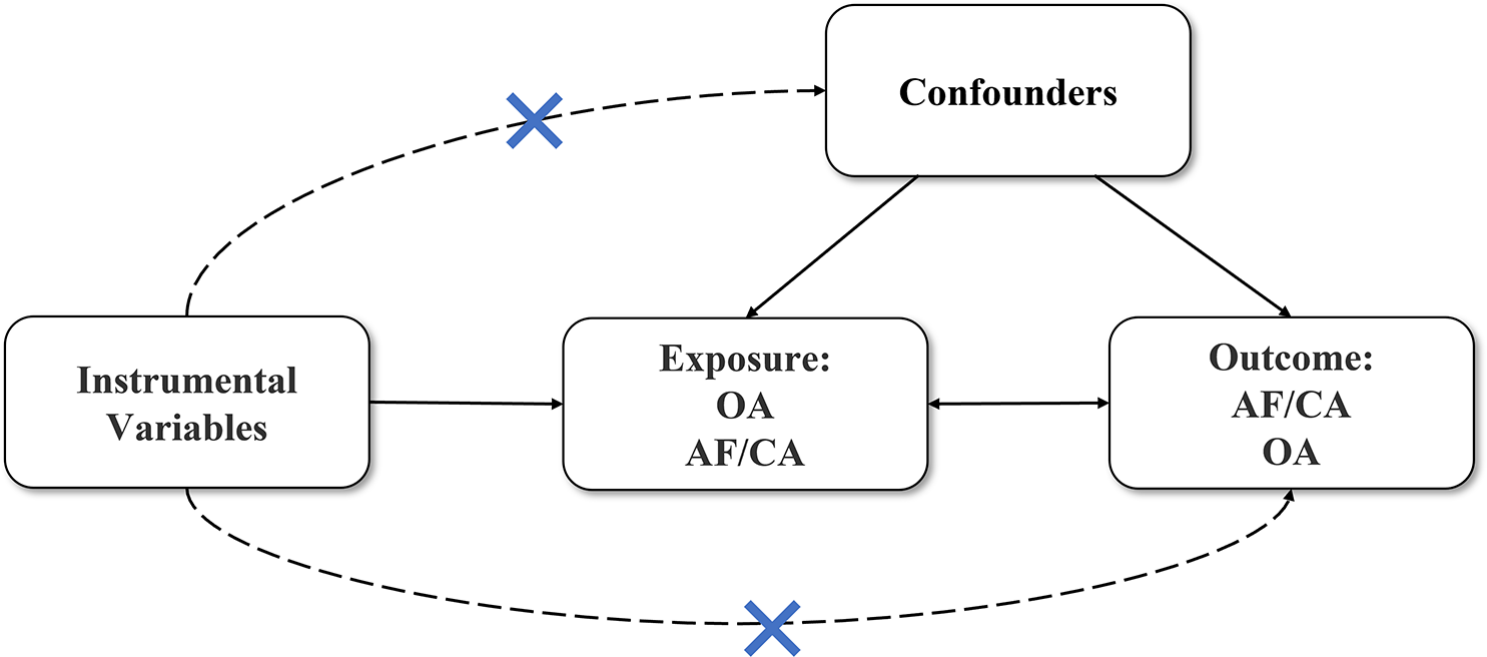
Main process of the study.

We used a bidirectional two-sample Mendelian randomization method to confirm whether there is a causal association between OA with AF and CA. In order to ensure the results were reliable, the MR analysis had to meet the following three key assumptions: (1) the genetic effects in the analysis have a very strong association with exposure; (2) the instrumental variables, as exposure factors, should affect the outcome via the exposure factors we studied exclusively; and (3) Instrumental variables should be independent of confounders of exposure and outcome (16). Single nucleotide polymorphisms (SNPs) that strictly met the above three conditions were considered as iv (17). Figure 1 shows the primary description of the method.

### 2.2. Data sources

The OA data were obtained from a combination of UK Biobank and arcOGEN resources, including 39,427 cases of knee or hip osteoarthritis and a control sample of 378,169 (18).

Data on atrial fibrillation were obtained from a total of 60,620 cases of European ancestry and 970,216 controls from six contributing studies (The Nord-Trøndelag Health Study (HUNT), deCODE, the Michigan Genomics Initiative (MGI), DiscovEHR, UK Biobank, and the AFGen Consortium) (19).

Coronary atherosclerosis data were obtained from FinnGen (https://www.finngen.fi). 23,363 cases and 195,429 controls were included (20).

All data derived from publicly available GWAS data, which are available on the Integrative Epidemiology Unit (IEU) GWAS database (https://gwas.mrcieu.ac.uk), with no need for informed consent of the participant. Table 1 provides specific information on all materials.

**Table 1:** The contribution research description of Mendelian randomization.

Type	Population	Gender	Number of SNPs	Simple size	Cases	Controls	Access address	DOI
OA	Europeans	NA	30,265,359	417,596	39,427	378,169	https://gwas.mrcieu.ac.uk/datasets/ebi-a-GCST007092/	10.1038/s41588-018-0327-1
AF	Europeans	NA	33,519,037	1,030,836	60,620	970,216	https://gwas.mrcieu.ac.uk/datasets/ebi-a-GCST006414/	10.1038/s41588-018-0171-3
CA	Europeans	Males and Females	16,380,466	218,792	23,363	195,429	https://gwas.mrcieu.ac.uk/datasets/finn-b-I9_CORATHER_EXNONE/	10.1101/2022.03.03.22271360

SNP, single nucleotide polymorphism; OA, osteoarthritis; AF, atrial fibrillation; CA, coronary atherosclerosis.

### 2.3. SNP selection

For each SNP independent variable, defined as those without linkage disequilibrium, we set a genome-wide significance threshold *P*-value (*P* < 5 × 10^−8^), a linkage disequilibrium correlation coefficient *r*^2^ (*r*^2^ < 0.001), and the number of bases between two SNPs (kb > 10,000), with further quality control based on minor allele frequency >1%. Finally, 24 OA-associated SNPs (Table 2), 23 CA-associated SNPs (Table 3), and 108 AF-associated SNPs were extracted respectively (Supplementary File S1). In Mendelian randomization studies, weak instrumental variable bias is a very important issue. Weak instrumental variables are genetic variants that have low explanatory power for exposure. To avoid the presence of this bias, the F-statistic was introduced to assess this effect. No SNP with F (calculated as $F=\;\beta_{exposure}^{2}/SE_{exposure}^{2}$) <10 were found in the results (21).

**Table 2:** Osteoarthritis instrumental variables and F-statistics.

SNP	Chr	Pos	Effect_Allele	Other_Allele	Samplesize	Beta	SE	EAF	*P*-value	F*
rs2622873	1	10,34,66,053	C	T	417,596	−0.0684	0.0113	0.1290	1.581 × 10^−09^	36.64
rs2820443	1	21,97,53,509	C	T	417,596	0.0543	0.0083	0.2985	6.0104 × 10^−11^	42.80
rs4630744	2	33,46,1,375	G	A	417,596	−0.0538	0.0076	0.4916	2.0999 × 10^−12^	50.11
rs3821262	2	70,72,1,006	G	A	417,596	−0.0554	0.0076	0.4740	3.5188 × 10^−13^	53.14
rs12470967	2	19,26,71,981	G	A	417,596	−0.0480	0.0085	0.5744	1.534 × 10^−08^	31.89
rs3774354	3	52,81,7,675	A	G	417,596	0.0536	0.0079	0.3604	1.3671 × 10^−11^	46.03
rs11923760	3	13,17,85,490	G	C	417,596	−0.0448	0.0082	0.3209	4.158 × 10^−08^	29.85
rs11732213	4	17,04,244	C	T	417,596	−0.0588	0.0096	0.1945	8.8139 × 10^−10^	37.52
rs3884606	5	17,08,71,074	A	G	417,596	−0.0437	0.0076	0.5119	8.2459 × 10^−09^	33.06
rs9277552	6	33,05,5,501	T	C	417,596	−0.0592	0.0093	0.2098	2.366 × 10^−10^	40.52
rs10948196	6	44,959,384	T	A	417,596	0.0426	0.0078	0.3861	4.496 × 10^−08^	29.83
rs2299285	7	95,71,1,902	A	G	417,596	0.0463	0.0080	0.3434	7.5669 × 10^−09^	33.50
rs11997261	8	85,24,361	C	T	417,596	−0.0542	0.0087	0.2819	5.158 × 10^−10^	38.81
rs4979341	9	11,69,05,543	T	C	417,596	0.0597	0.0086	0.2697	3.3527 × 10^−12^	48.19
rs10758594	9	42,95,583	G	A	417,596	0.0436	0.0077	0.5844	1.686 × 10^−08^	32.06
rs17659798	11	28,87,4,997	C	A	417,596	−0.0539	0.0085	0.2869	2.058 × 10^−10^	40.21
rs7935877	11	65,34,5,765	T	C	417,596	−0.0822	0.0149	0.0710	3.407 × 10^−08^	30.43
rs10492367	12	28,01,4,970	T	G	417,596	0.0545	0.0097	0.1895	1.959 × 10^−08^	31.57
rs4144502	12	94,18,1,328	A	G	417,596	0.0468	0.0076	0.5118	9.48 × 10^−10^	37.92
rs56116847	12	12,38,35,233	A	G	417,596	0.0453	0.0080	0.3564	1.282 × 10^−08^	32.06
rs2472304	15	75,04,4,238	A	G	417,596	0.0452	0.0081	0.6680	2.025 × 10^−08^	31.14
rs2953013	17	29,49,6,343	A	C	417,596	−0.0524	0.0083	0.7048	3.065 × 10^−10^	39.86
rs75621460	19	41,83,3,784	A	G	417,596	0.1523	0.0256	0.0267	2.88 × 10^−09^	35.39
rs143384	20	34,02,5,756	G	A	417,596	−0.0634	0.0077	0.4035	2.4249 × 10^−16^	67.79
rs9977881	21	40,00,7,222	C	T	417,596	0.0607	0.0102	0.1695	2.543 × 10^−09^	35.41

SNP, single nucleotide polymorphism; Beta, the effect size; SE, standard error; EAF, effect allele frequency.

* F-statistics = 38.63.

**Table 3:** Coronary atherosclerosis instrumental variables and F-statistics.

SNP	Chr	Pos	Effect_Allele	Other_Allele	Samplesize	Beta	SE	EAF	Pval	F*
rs11591147	1	55,50,5,647	T	G	218,792	−0.2204	0.0337	0.0361	6.33 × 10^−11^	42.77
rs629301	1	10,98,18,306	T	G	218,792	0.1032	0.0152	0.7853	1.07 × 10^−11^	46.10
rs72661887	1	38,41,6,310	T	C	218,792	0.0777	0.0125	0.5369	4.55 × 10^−10^	38.64
rs72664324	1	56,97,2,353	A	G	218,792	−0.1089	0.0198	0.1107	4.05 × 10^−08^	30.25
rs4835377	4	14,80,39,045	G	A	218,792	−0.1057	0.0150	0.7774	1.71 × 10^−12^	49.66
rs59415853	4	14,83,78,225	A	G	218,792	0.1132	0.0185	0.1275	1.04 × 10^−09^	37.44
rs9285863	5	10,80,71,655	C	T	218,792	−0.0815	0.0134	0.3137	1.26 × 10^−09^	36.99
rs9349379	6	12,90,3,957	G	A	218,792	0.1335	0.0124	0.4507	7.63 × 10^−27^	115.91
rs34537042	6	13,40,17,855	A	G	218,792	0.0886	0.0161	0.1830	3.38 × 10^−08^	30.28
rs117733303	6	16,09,22,870	G	A	218,792	0.5536	0.0605	0.0113	5.73 × 10^−20^	83.73
rs10455872	6	16,10,10,118	G	A	218,792	0.3289	0.0300	0.0458	5.10 × 10^−28^	120.19
rs12705390	7	10,64,10,777	A	G	218,792	0.0802	0.0135	0.2981	2.95 × 10^−09^	35.29
rs3918226	7	15,06,90,176	T	C	218,792	0.1353	0.0243	0.0699	2.70 × 10^−08^	31.00
rs9644861	9	22,09,0,935	T	C	218,792	0.2062	0.0125	0.4183	9.89 × 10^−61^	272.12
rs964184	11	11,66,48,917	C	G	218,792	−0.1079	0.0175	0.8542	6.90 × 10^−10^	38.02
rs653178	12	11,20,07,756	T	C	218,792	−0.0773	0.0126	0.5840	8.12 × 10^−10^	37.64
rs750597	13	11,10,29,256	A	T	218,792	−0.0809	0.0133	0.3204	1.22 × 10^−09^	37.00
rs118092637	14	10,00,58,036	C	T	218,792	0.3029	0.0470	0.0183	1.17 × 10^−10^	41.53
rs11852887	15	79,03,0,578	C	A	218,792	−0.0959	0.0129	0.6411	1.20 × 10^−13^	55.27
rs191156695	15	89,44,7,985	T	C	218,792	−0.2902	0.0378	0.0285	1.75 × 10^−14^	58.94
rs113113862	19	11,18,3,577	A	G	218,792	−0.1006	0.0152	0.2147	3.51 × 10^−11^	43.80
rs7412	19	45,41,2,079	T	C	218,792	−0.1966	0.0278	0.0535	1.40 × 10^−12^	50.01
rs28451064	21	35,59,3,827	A	G	218,792	0.1089	0.0173	0.1534	2.74 × 10^−10^	39.62

SNP, single nucleotide polymorphism; Beta, the effect size; SE, standard error; EAF, effect allele frequency.

* F-statistics = 59.66.

### 2.4. Statistical analyses

On the basis of the above data, we used the two-sample MR method to analyze the relationship between OA with AF and CA. The main analysis method used was inverse variance weighted (IVW) analysis, which assumed that all SNPs are valid instrumental variables and provide the most accurate estimates. The results obtained by IVW had high statistical power when all SNPs met the three main hypotheses (22). Meanwhile, to complement the results, four other methods were selected (MR Egger, weighted median, simple model, and weighted model) (23). Median estimates include weighted median, simple mode, and weighted mode. The weighted median method can provide consistent estimates when more than 50% of the weight comes from valid instrument variants. MR-Egger regression is able to generate estimates after accounting for horizontal pleiotropy, but with a lower degree of precision.

To make sure that the results were accurate, we used the Cochran's Q test to evaluate whether there was heterogeneity between the individual SNPs, with a *P*-value <0.05 judging the presence of heterogeneity in the results. MR Egger analysis was used to assess the multiplicity of SNPs, which can be considered absent if *P* > 0.05. In addition, leave-one-out analysis was conducted to determine if there was an instrumental variable with more influence on the results. The R package (TwoSampleMR) in R version 4.2.2 was used for all analyses.

## 3. Results

### 3.1. The causal effect between OA and AF

**Figure 2:**
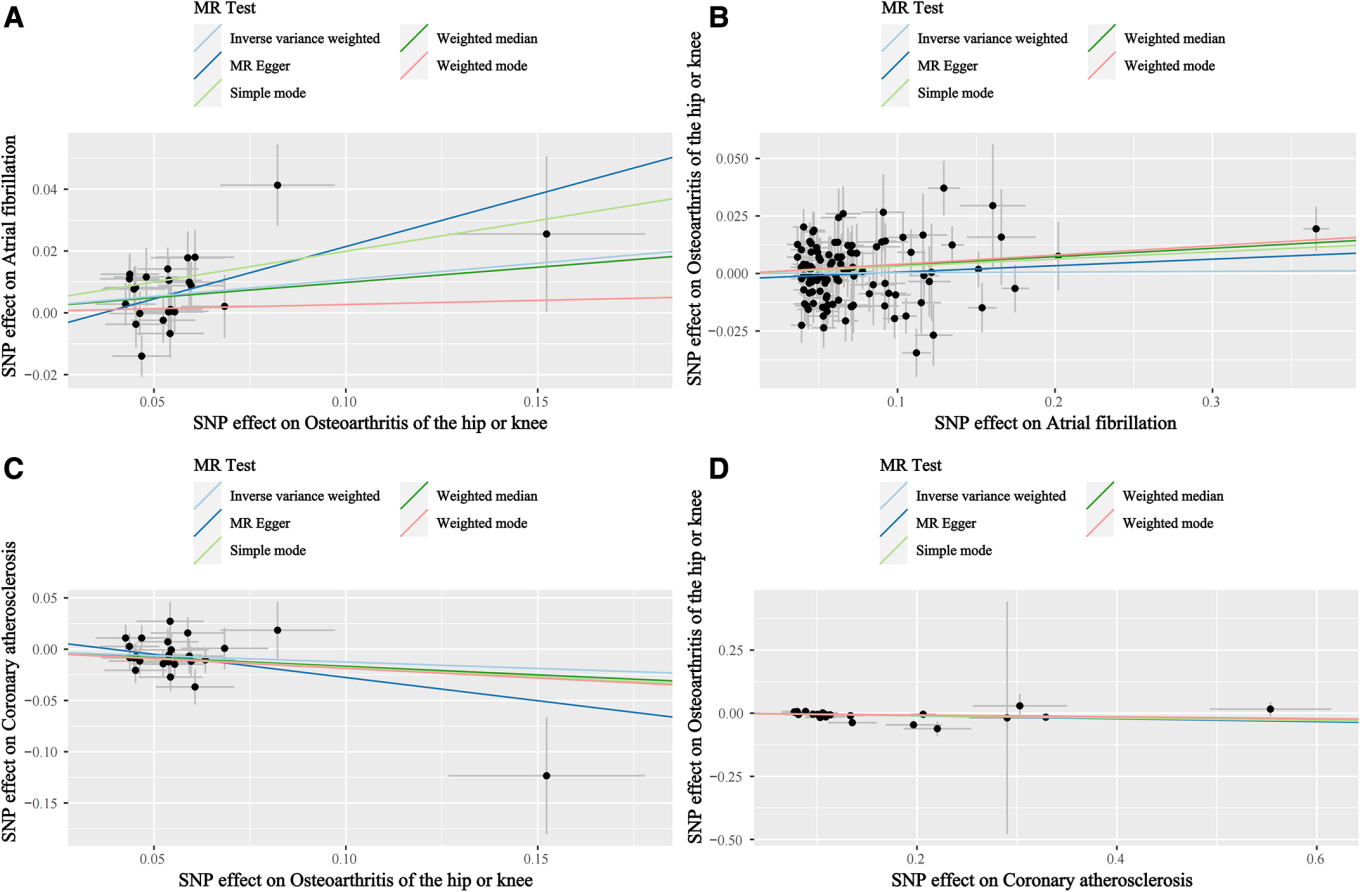
Scatterplots. (**A**) Scatterplots of the causal relationships between osteoarthritis and atrial fibrillation. (**B**) Scatterplots of the causal relationships between atrial fibrillation osteoarthritis and osteoarthritis. (**C**) Scatterplots of the causal relationships between osteoarthritis and coronary atherosclerosis. (**D**) Scatterplots of the causal relationships between coronary atherosclerosis and coronary atherosclerosis osteoarthritis.

**Figure 3:**
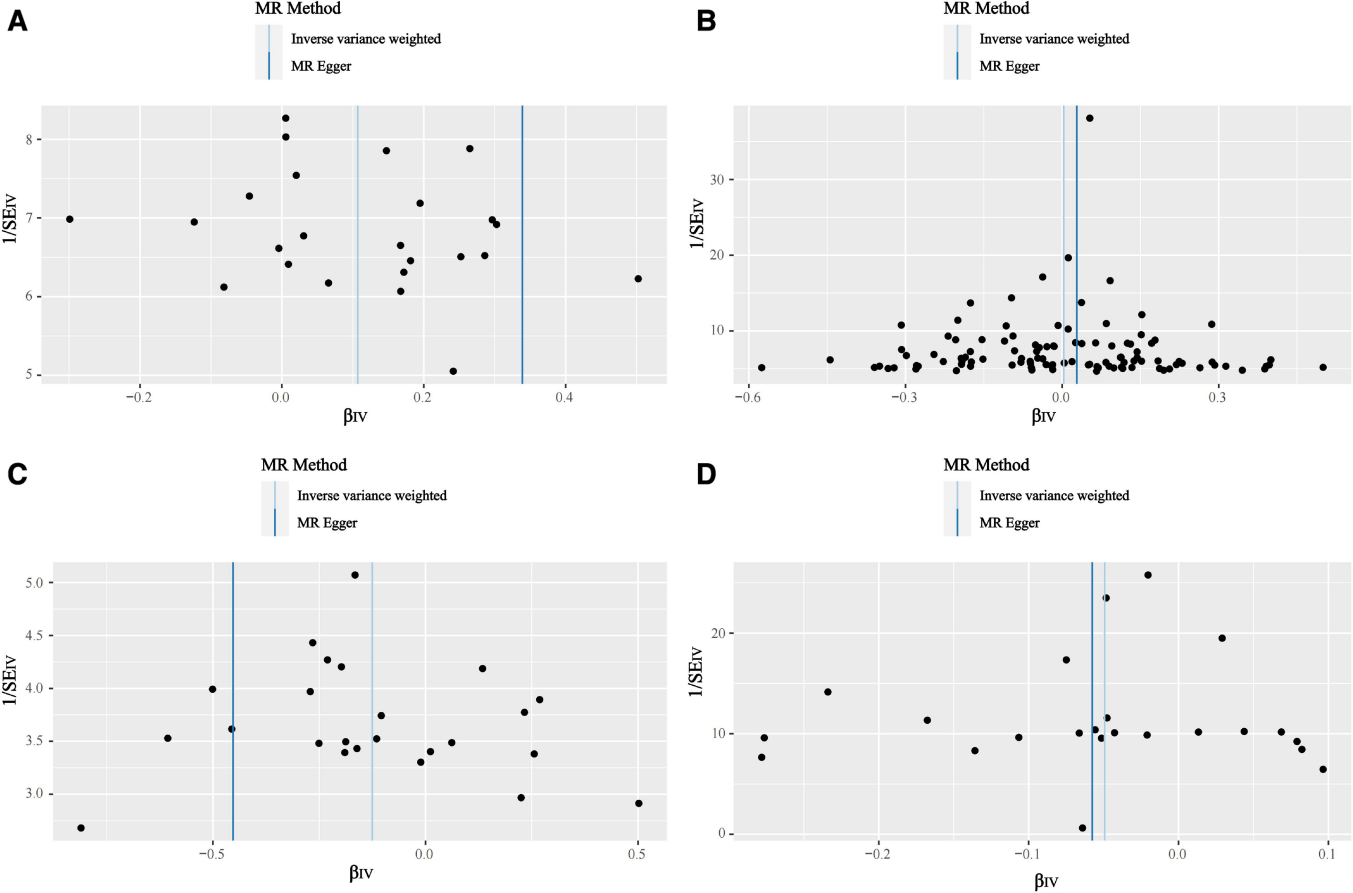
Funnel plot. (**A**) Funnel plot of the causal relationships between osteoarthritis and atrial fibrillation. (**B**) Funnel plot of the causal relationships between atrial fibrillation osteoarthritis and osteoarthritis. (**C**) Funnel plot of the causal relationships between osteoarthritis and coronary atherosclerosis. (**D**) Funnel plot of the causal relationships between coronary atherosclerosis and coronary atherosclerosis osteoarthritis.

**Figure 4:**
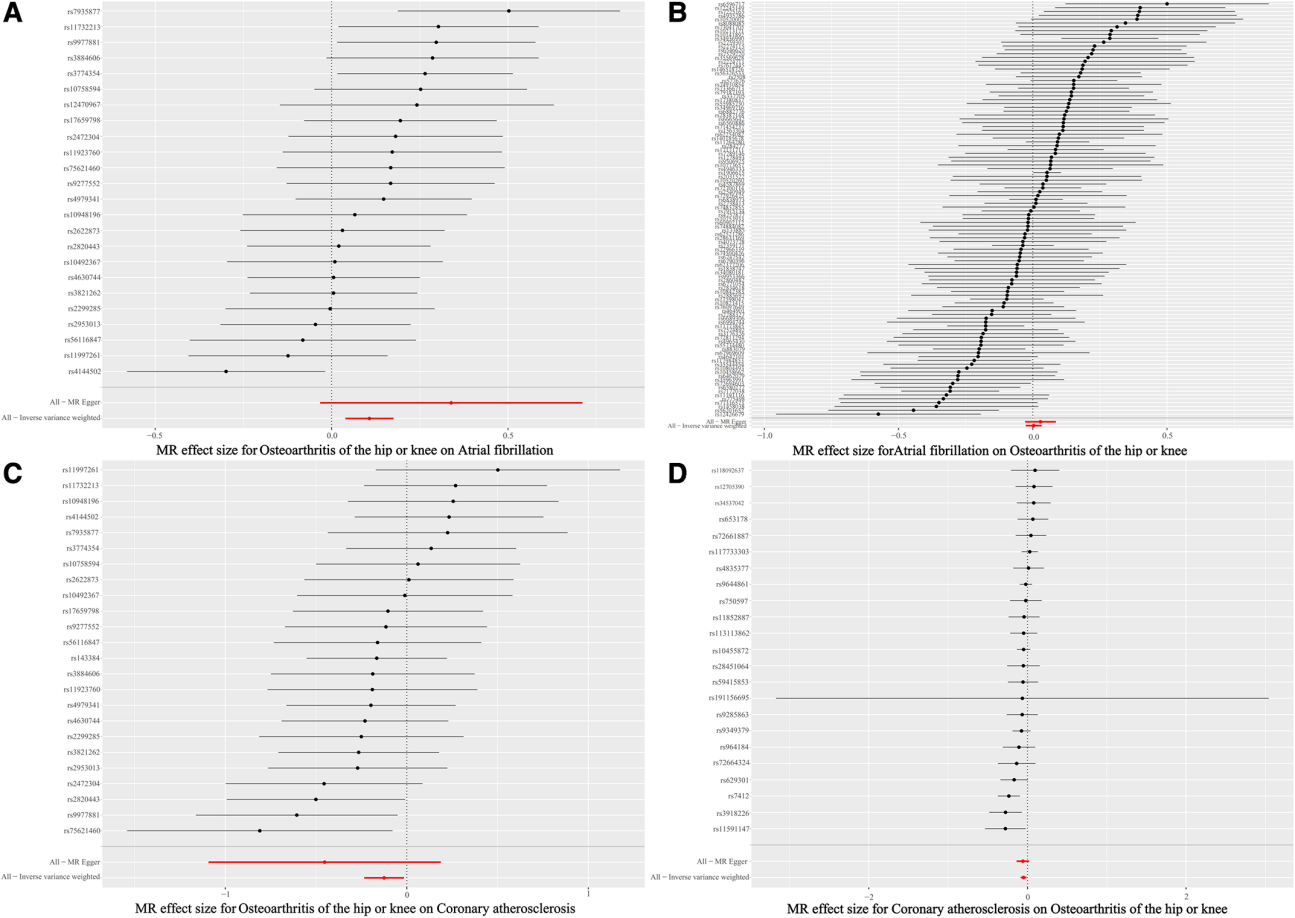
Single SNP effect combination forest plot. (**A**) Single SNP effect combination forest plot of the causal relationships between osteoarthritis and atrial fibrillation. (**B**) Single SNP effect combination forest plot of the causal relationships between atrial fibrillation osteoarthritis and osteoarthritis. (**C**) Single SNP effect combination forest plot of the causal relationships between osteoarthritis and coronary atherosclerosis. (**D**) Single SNP effect combination forest plot of the causal relationships between coronary atherosclerosis and coronary atherosclerosis osteoarthritis.

**Figure 5:**
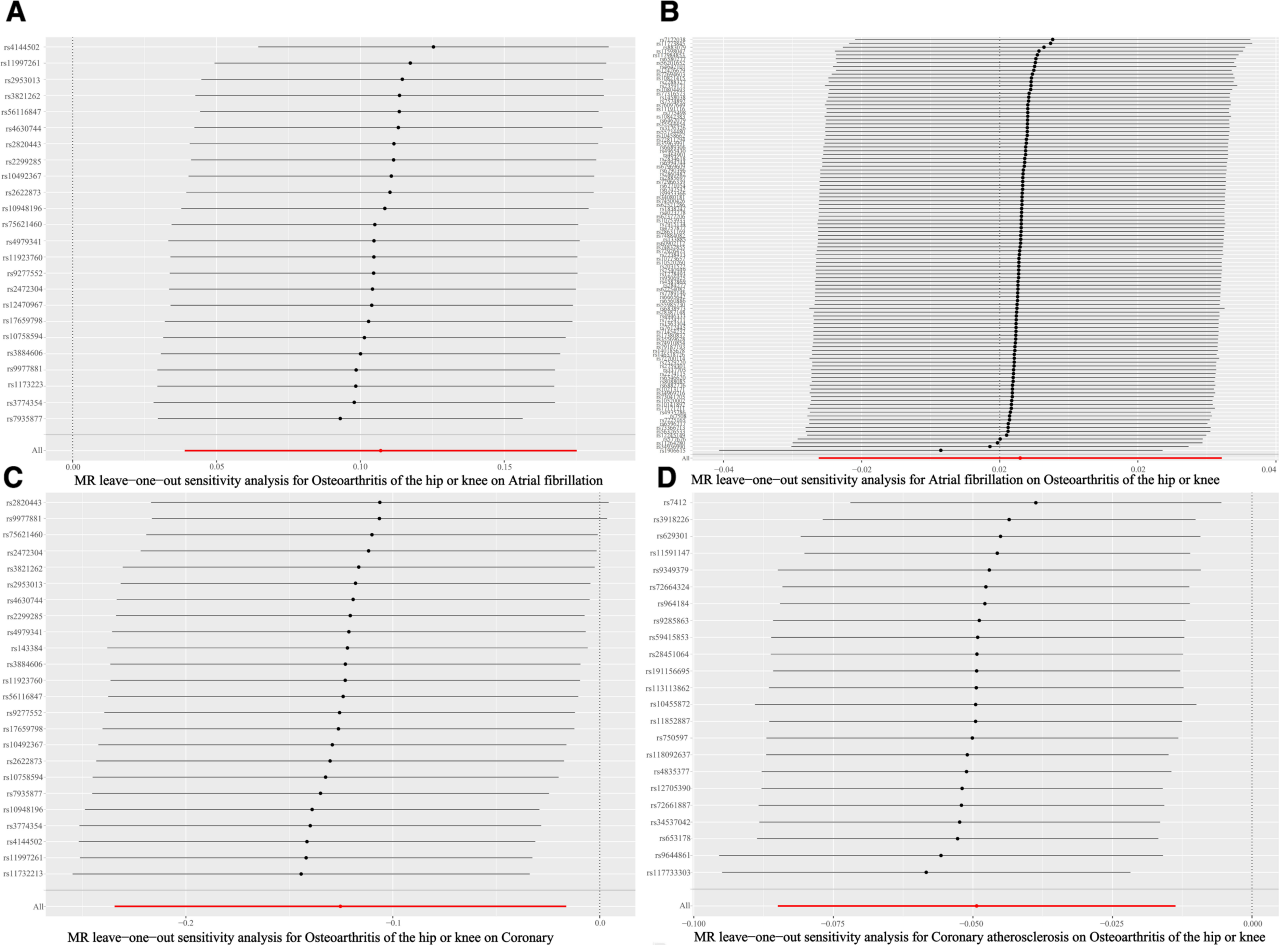
Forest plot of leave-one-out analysis. (**A**) Forest plot of leave-one-out analysis of the causal relationships between osteoarthritis and atrial fibrillation. (**B**) Forest plot of leave-one-out analysis of the causal relationships between atrial fibrillation osteoarthritis and osteoarthritis. (**C**) Forest plot of leave-one-out analysis of the causal relationships between osteoarthritis and coronary atherosclerosis. (**D**) Forest plot of leave-one-out analysis of the causal relationships between coronary atherosclerosis and coronary atherosclerosis osteoarthritis.

Based on the results of the IVW shown in Table 4, we found that OA increased the incidence of AF (IVW: OR (95% CI): 1.11 (1.04, 1.19), *P* = 0.002), which was consistent with the results of the Weighted median and Simple mode (Table 4). The results of the heterogeneity and pleiotropy tests were presented in Table 5, with *P* > 0.05, which could indicate that there was no heterogeneity or pleiotropy in the study. The scatter plot can visualize the positive associations between OA and CA (Figure 2A). The funnel plot (Figure 3A) is symmetric and consistent with the results of the heterogeneity analysis. The Forest plot of the combination of single SNP effects could be found in Figure 4A. According to the results of the leave-one-out analysis (Figure 5A), no SNPs with significant effects could be identified, which showed the stability of the results. Therefore, we were able to consider OA as a risk factor for AF.

**Table 4:** Results of Mendelian randomization studies.

Exposure- outcome	nSNP	MR egger		Weighted median		Inverse variance weighted		Simple mode		Weighted mode	
		OR (95% CI)	*P*-value	OR (95% CI)	*P*-value	OR (95% CI)	*P*-value	OR (95% CI)	*P-*value	OR (95% CI)	*P-*value
OA-AF	24	1.40 (0.97, 2.03)	0.09	1.10 (1.01, 1.20)	0.02	1.11 (1.04, 1.19)	0.00	1.22 (1.02, 1.46)	0.03	1.03 (0.87, 1.21)	0.76
AF-OA	108	1.03 (0.97, 1.09)	0.33	1.04 (1.00, 1.08)	0.06	1.00 (0.97, 1.03)	0.84	1.03 (0.93, 1.14)	0.55	1.04 (1.00, 1.09)	0.08
OA-CA	24	0.64 (0.34, 1.21)	0.18	0.85 (0.73, 0.99)	0.03	0.88 (0.79, 0.98)	0.02	0.84 (0.63, 1.12)	0.24	0.83 (0.64, 1.07)	0.17
CA-OA	23	0.94 (0.87, 1.02)	0.17	0.95 (0.91, 1.00)	0.05	0.95 (0.92, 0.99)	0.01	0.95 (0.89, 1.03)	0.22	0.97 (0.92, 1.02)	0.20

SNP, single nucleotide polymorphism; OR, odds ratio; CI, Confidence interval; OA, osteoarthritis; AF, atrial fibrillation; CA, coronary atherosclerosis.

**Table 5:** Results of heterogeneity analysis and pleiotropy.

Exposure- outcome	nSNP	Heterogeneity (Q_*P*-value)		Pleiotropy (*P-*value)
		IVW	MR egger	Egger_Intercept
OA-AF	24	0.11	0.14	0.23
AF-OA	108	5.46 × 10^−06^	4.91 × 10^−06^	0.31
OA-CA	24	0.43	0.43	0.32
CA-OA	23	0.22	0.18	0.82

SNP, single nucleotide polymorphism; OA, osteoarthritis; AF, atrial fibrillation; CA, coronary atherosclerosis; MR, Mendelian randomization; IVW, inverse variance weighting.

In contrast, AF has no effect on OA. The results were shown in Table 4, with *P*-values >0.05 for all five statistical methods.

### 3.2. The causal effect between OA and Ca

The results of the five statistical methods for the assessment of the causal relationship between OA and CA were shown in Table 4, indicating that OA was a protective factor for CA (IVW: OR (95% CI): 0.88 (0.79, 0.98), *P *= 0.02; Weighted median: OR (95% CI): 0.85 (0.73, 0.99), *P *= 0.03). *P*-values for heterogeneity and pleiotropy were >0.05 (Table 5), indicating that there was no heterogeneity or pleiotropy in the study. Scatter plots could be found in Figure 2C, forest plots of the combination of single SNP effects in Figure 4C, funnel plots in Figure 3C, and leave-one-out analysis plots in Figure 5C. The results were stable, and it could be considered that OA was a protective factor for CA.

Meanwhile, a negative associations was observed between CA and OA (Table 4). The results showed no heterogeneity or pleiotropy (Table 5), and according to the leave-one-out analysis results (Figure 5D), the results were stable.

## 4. Discussion

Using the genetic data in the open database, we performed an MR analysis of the association between OA with AF and CA. According to the results, OA and AF are positively associated, but no effect of AF on OA, while OA and CA are negatively associated with each other, in agreement with the results of some observational studies. A retrospective electronic medical record review from the United States found that osteoarthritis, a common comorbidity of atrial fibrillation, significantly increased the incidence of atrial fibrillation. The total number of cases was 2,433, of which 418 had AF and 216 also had OA (51.7%, *P* < 0.001) (24). Cross-sectional findings show a small negative association between HOA and cardiovascular events despite increased vascular pathology (25). The study included 2,264 men (mean age 76 years; SD 6) and 3,078 women (mean age 76 years; SD 6). Our study is the first to confirm a causal relationship between OA with AF and CA from a genetic perspective.

At present, the mechanisms of association between the three diseases are unclear. However, the causal relationship can be explained in the following ways. First, there are a number of risk factors in common between OA, AF and CA, such as advanced age and obesity, both of which contribute to the incidence of osteoarthritis and cardiovascular disease. Second, the use of NSAIDs (non-steroidal antiinflammatory drugs) for pain relief is more common in OA, which is also found to be related to an increasing prevalence of CVD (26). The primary mechanism by which NSAIDs cause heart failure is thought to be inhibition of renal prostaglandin synthesis, resulting in afferent small artery vasoconstriction and sodium and fluid retention. Thus, NSAIDs may counteract the physiological mechanisms by which the kidney compensates for impaired cardiac output (27). In contrast, the use of coronary medications leads to a decrease in the incidence of osteoarthritis. While angiotensin-aldosterone system modulators (captopril, enalapril, cloxazine) have both stimulatory and inhibitory effects on cartilage regeneration, beta-adrenergic receptor inhibitors may reduce the negative effects of the adrenergic system on cartilage formation, such as reduced ECM production (28). In addition, the painful symptoms of osteoarthritis have a deleterious effect not only on sedentary behavior, but also on physical activity levels, which are significant risk factors for atrial fibrillation (29). On the contrary, coronary atherosclerosis also reduces the amount of physical activity, which in turn may reduce the load on the knee and hip joints, thereby reducing the incidence of osteoarthritis (30).

The common mechanisms among the three diseases can be analyzed at the molecular level, mainly considering genetic mechanisms and inflammation. From a genetic point of view, a cohort study from the Framingham Offspring Study found that the risk of atrial fibrillation in offspring with at least one parent with atrial fibrillation was 1.85 times higher than without atrial fibrillation, increasing to 3.23 times after adjustment for age of onset of atrial fibrillation below 75 years (31). Familial atrial fibrillation is considered a major risk factor independent of other conventional risk factors (32). MiRNAs can also affect many genes associated with atrial fibrillation, making them viable molecular targets. For example, miR-21 and miR-133 may participate in structural remodeling of the atria through increased fibrosis (33). The Stockholm-Tartu Atherosclerosis Reverse Network Engineering Task (STARNET) study identified 224 gene-regulatory co-expression networks that could explain 54% of CAD heritability (34). A twin study confirmed a significant influence of genetic factors in women with hip osteoarthritis (35). Genetic factors are among the risk factors for all three diseases. There may be common genetic mechanisms, some of which are unknown to us.

Studies have shown that fibrosis is essential for the development of atrial fibrillation and that fibroblasts are important cytokines for atrial fibrosis (36). In particular, a biochemical signal, TGF-β, which is important to induce fibroblast differentiation, mediates the transcription of myofibroblast genes (37). In addition, mitochondria play a key regulatory role in fibroblast activation (38). In the pathogenesis of OA, the mitochondrial pathway has also been implicated in the apoptosis of chondrocytes induced by mechanical stress (39). TGF-β generally plays a chondroprotective role, but under some circumstances it also determines OA-like changes in healthy articular cartilage by promoting the differentiation and apoptosis of terminal chondrocytes (40). We speculate that there may be pathways that operate simultaneously in the development of both OA and AF. In addition, inflammation, which contributes to atrial remodeling and is associated with the occurrence and maintenance of atrial fibrillation, is an essential pathogenic mechanism in both osteoarthritis and atrial fibrillation. Results of a large prospective study suggest that inflammation is a powerful predictor of AF (41). It has been shown that NLRP3 inflammatory vesicle activity is significantly elevated in cardiomyocytes from patients with atrial fibrillation, which has implications for cardiac fibroblast activation (36). Significant inflammatory factors such as IL-6 and CRP have also been independently found to correlate with the incidence of AF (42).

AF, CA, and OA are all highly prevalent in the population and are often referred to as comorbidities (43). AF has a severe effect on the quality of life and is often associated with serious complications that lead to increased mortality, reduced quality of life, and expensive medical costs (44). Confirmation of a causal relationship between OA with AF and CA may help to prevent and manage the disease by screening high-risk groups, thereby reducing morbidity or delaying the disease process. For example, regular physical examinations in patients with osteoarthritis can detect the presence of cardiovascular disease at an early stage. In addition, health education efforts can be made to reduce the incidence of cardiovascular disease through lifestyle changes in patients with osteoarthritis.

The strength of this study is that it confirms for the first time the causal relationship between OA with AF and CA from a genetic perspective, avoiding the potential bias of observational studies. However, there are some limitations to this study as well. First, all data were obtained from the European population, which needs further validation to generalize to other populations. Second, the data were not stratified by sex, age, or disease severity. Nevertheless, the SNPs included in this study all meet the requirements for valid instrumental variables, so our results are reliable.

## 5. Conclusion

This study confirmed that OA leads to an increased prevalence of AF and decreased prevalence of CA by a bidirectional two-sample Mendelian randomization study. Although the results need to be validated by further clinical and basic experiments, they provide a new perspective for future research on the potential link between OA and CVD.

## Data Availability

The original contributions presented in the study are included in the article/**Supplementary Material**, further inquiries can be directed to the corresponding authors.
